# Looking Beyond the Surface: A Case of Shigella Enterocolitis in a Patient Presenting With Weakness and Substance Use

**DOI:** 10.7759/cureus.102393

**Published:** 2026-01-27

**Authors:** Erika Lytle, Kevin Le, Huan Le

**Affiliations:** 1 Internal Medicine, University of South Florida Morsani College of Medicine, Tampa, USA; 2 Internal Medicine, Edward Via College of Osteopathic Medicine, Shreveport, USA; 3 Internal Medicine, Willis Knighton Health, Shreveport, USA

**Keywords:** enterocolitis, infectious colitis, shigella, shigella colitis, shigellosis

## Abstract

*Shigella* species are common causes of enteric infection, yet atypical presentations can complicate diagnosis in adults. Rising antimicrobial resistance further heightens the need for prompt and accurate identification. Atypical or non-gastrointestinal presentations remain underrecognized and risk diagnostic delay.

We describe a 46-year-old man with no medical history who presented with fatigue, abdominal pain, and diarrhea following methamphetamine use. He was febrile and tachycardic with leukocytosis and elevated lactate. CT imaging demonstrated acute colitis, and stool cultures confirmed *Shigella sonnei*. He improved with empiric intravenous piperacillin-tazobactam and was transitioned to oral levofloxacin based on susceptibilities.

This case illustrates the diagnostic challenges posed by unreliable histories, substance use, and non-specific presentations. Early imaging and stool diagnostics were key to timely management. As antimicrobial resistance among *Shigella* species continues to rise, maintaining a broad differential and guiding therapy with culture and susceptibility data remains essential.

## Introduction

*Shigella* species are enteroinvasive, Gram-negative bacteria transmitted via the fecal-oral route, directly through person-to-person contact, and indirectly through contaminated food and water [1]. The genus comprises four major subgroups - *Shigella dysenteriae* (group A), *Shigella flexneri* (group B), *Shigella boydii* (group C), and *Shigella sonnei* (group D) - each with distinct epidemiologic characteristics [2]. In the United States, *S. sonnei* is the predominant serogroup, accounting for nearly 80% of bacillary dysentery cases [3]. Clinical features of shigellosis, an acute enteric infection caused by *Shigella* species, typically include abdominal pain, fever, tenesmus, and mucoid or bloody diarrhea [1,4]. Severe disease is more commonly recognized in high-risk populations such as children, immunocompromised individuals, and adults with behavioral or social risk factors, including substance use or limited healthcare access [1]. In contrast, immunocompetent adults without these risk factors may present atypically, with absent or subtle gastrointestinal symptoms, which can lead to delayed recognition and suboptimal treatment [1].

Despite recognition of these high-risk groups, there is a gap in the literature regarding atypical presentations in otherwise healthy adults and strategies to ensure timely diagnosis. Current clinical interventions to address shigellosis include early stool culture for pathogen identification, empiric antibiotic therapy guided by local resistance patterns, and antimicrobial stewardship practices, including prompt de-escalation based on susceptibility testing [4-6].

Our patient, an immunocompetent adult, presented with vague systemic symptoms - generalized weakness, nonproductive cough, and diffuse abdominal pain - without classic dysenteric features. The initial presentation was further complicated by recent methamphetamine use, which obscured history-taking and may have influenced early clinical assessment.

The objective of this case report is to highlight this atypical presentation of *Shigella sonnei *infection in an adult without classic gastrointestinal symptoms. By describing this case, we aim to underscore the need for heightened clinical vigilance, a broad differential diagnosis for systemic symptoms in adults, and early diagnostic evaluation, particularly in the context of rising antimicrobial resistance and changing epidemiologic patterns of shigellosis.

## Case presentation

A 46-year-old Caucasian male with no known medical history presented to the emergency department (ED) complaining of generalized weakness, nonproductive cough, left-sided abdominal pain, and diarrhea, all beginning one day prior to presentation. History-taking was limited by the patient’s altered mentation and communication difficulties. A urine drug screen was positive for methamphetamine, though the timing of use relative to symptom onset was uncertain. The patient was unemployed, denied recent travel or sick contacts, and reported no high-risk food exposures, while remaining primarily within his local community.

**Figure 1:**
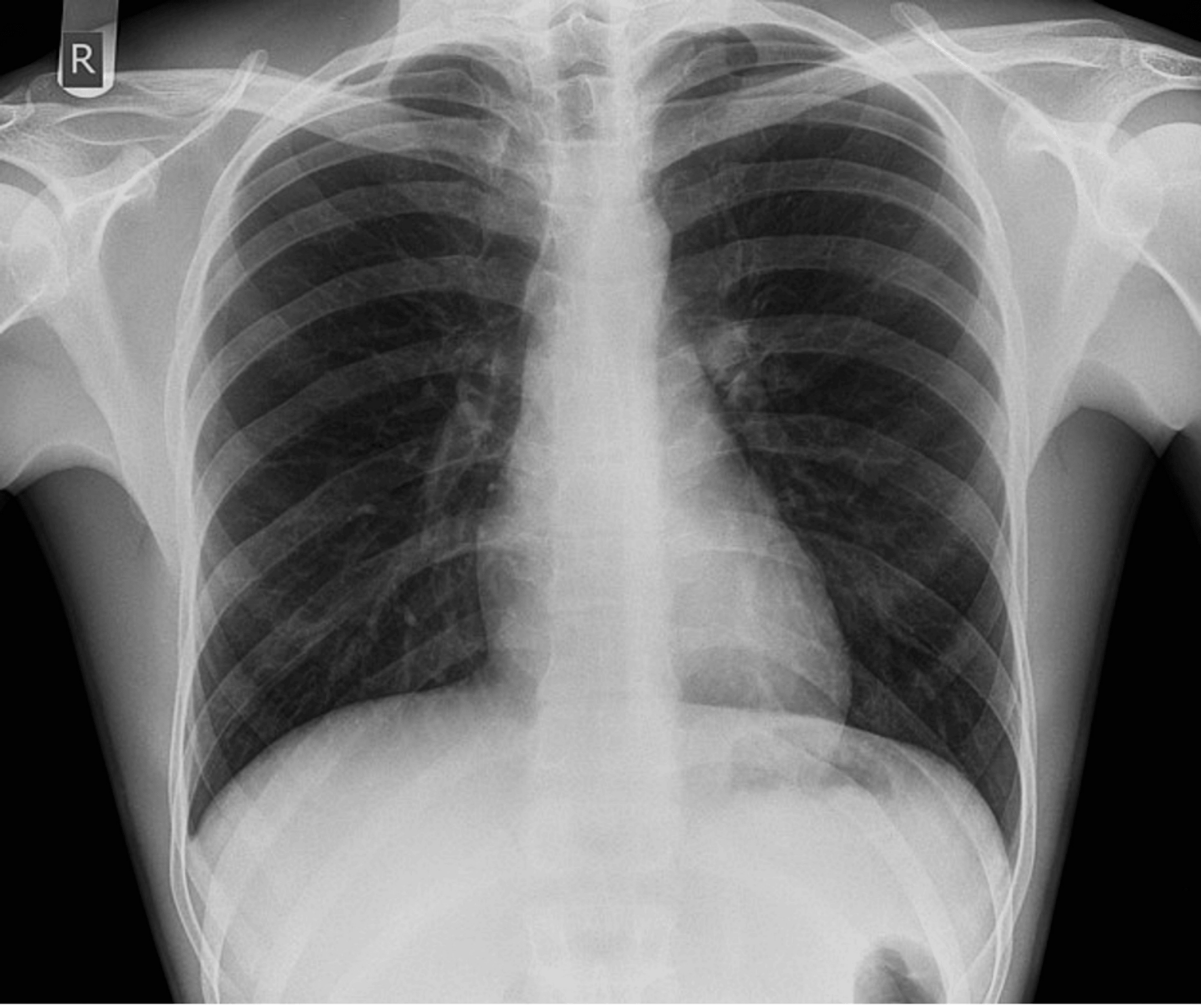
Normal Chest Radiograph at Presentation Patient’s chest X-ray demonstrating no evidence of cardiomegaly, pleural effusions, or pulmonary congestion.

Upon further questioning in the ED, the patient described the abdominal pain as mild and diffuse, rated 4/10 in intensity, without associated tenesmus or hematochezia, and reported three to four episodes of non-bloody diarrhea over the preceding 24 hours. Vital signs showed a low-grade fever of 38.1°C and mild tachycardia. Laboratory evaluation revealed mild leukocytosis (WBC 11.7 × 10^9^/L) and elevated lactate (3.9 mmol/L), indicating systemic inflammation and early metabolic stress. Other laboratory values, including a complete metabolic panel and HIV testing, were within normal limits (Table 1). Chest X-ray was unremarkable, ruling out concurrent pulmonary infection (Figure 1).

**Table 1:** Key Laboratory Values at Presentation Laboratory values obtained on presentation, including complete blood count and complete metabolic panel. The patient demonstrated mild leukocytosis and elevated lactate, while other parameters were within normal limits. Reference ranges are provided where applicable. AST: Aspartate transaminase; ALT: Alanine transaminase; BUN: Blood urea nitrogen

Laboratory Test	Patient Value	Reference Range	Units
White Blood Cell Count (WBC)	11.7	4.0–10.5	×10⁹/L
Hemoglobin	14.1	13.5–17.5	g/dL
Platelets	367	150–450	×10⁹/L
Lactate	3.9	0.5–2.2	mmol/L
Sodium	134	135–145	mmol/L
Potassium	3.5	3.5–5.0	mmol/L
Chloride	99	98–107	mmol/L
CO₂ (Bicarbonate)	23	22–29	mmol/L
BUN	18	7–20	mg/dL
Creatinine	0.9	0.6–1.3	mg/dL
AST	23	10–40	U/L
ALT	27	7–56	U/L
Alkaline Phosphatase	121	44–147	U/L
Total Bilirubin	0.8	0.1–1.2	mg/dL
HIV (antigen/antibody)	Non-reactive	Non-reactive	-

**Figure 2:**
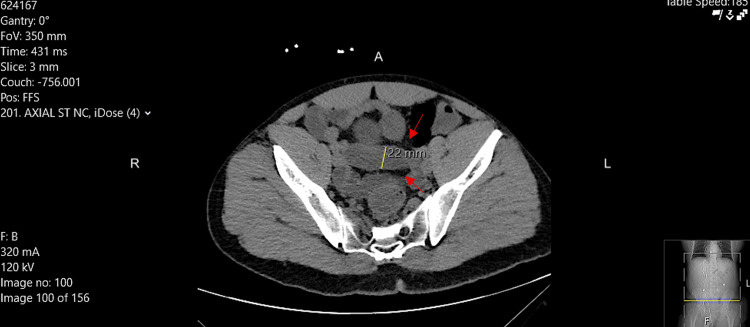
Axial Non-Contrast CT of the Abdomen and Pelvis Showing Colonic Inflammation Axial non-contrast CT of the abdomen and pelvis demonstrating segmental colonic wall thickening with mild pericolonic inflammatory changes (red arrows). The yellow line represents the measured colonic lumen diameter of 22 mm.

Although the patient's gastrointestinal symptoms were mild, the combination of fever, leukocytosis, and diffuse abdominal pain raised concern for a potentially serious intra-abdominal infection. Consequently, a non-contrast CT of the abdomen and pelvis was obtained, revealing segmental colonic wall thickening with mild pericolonic inflammatory changes, consistent with acute colitis (Figure 2). These laboratory and imaging findings guided the decision to initiate empiric intravenous piperacillin-tazobactam while awaiting stool culture results.

On hospital day 2, stool cultures grew *Shigella sonnei*, susceptible to levofloxacin, cefepime, and ertapenem. Antibiotic therapy was subsequently narrowed to oral levofloxacin 500 mg daily for five days. The patient demonstrated progressive clinical improvement, including resolution of fever and gastrointestinal symptoms, and tolerated advancement of diet without recurrence of symptoms. He was discharged on hospital day 6 in stable condition with oral antibiotics provided at discharge and instructions for outpatient follow-up. Follow-up was recommended to assess for symptom recurrence or complications; however, the patient did not attend scheduled outpatient visits.

Table 2 summarizes key atypical features, clinical significance, and comparisons with classic *Shigella* presentations, emphasizing systemic symptoms and immunocompetent status despite recent substance use.

**Table 2:** Comparison of Classic Shigella Presentation and Patient’s Atypical Features

Feature	Typical *Shigella* Presentation	Patient Presentation	Clinical Significance	Reference
Diarrhea	Frequent, mucoid or bloody	Mild, non-bloody, 3-4 episodes	Could lead to under-recognition if relying solely on classic dysenteric features	[1]
Tenesmus	Often present	Absent	Lack of tenesmus may delay consideration of enteric infection	[1]
Abdominal pain	Cramping, sometimes severe	Mild, diffuse	Mild pain may obscure severity of underlying colitis	[3]
Systemic symptoms	Fever, sometimes malaise	Fever, generalized weakness, nonproductive cough	Highlights that systemic signs can be key diagnostic clues	[3]
Risk Factors	Children, immunocompromised, high-risk behaviors	Immunocompetent adult, recent methamphetamine use	Highlights that even isolated behavioral risk factors can complicate assessment and contribute to atypical presentation	[3]
Laboratory Findings	Variable leukocytosis, mild metabolic stress	Mild leukocytosis (11.7 × 10^9^/L), elevated lactate (3.9 mmol/L)	Laboratory abnormalities prompted further imaging and empiric therapy	[3]
Imaging	Often not performed in mild cases	CT: segmental colonic wall thickening with mild pericolonic inflammation	Imaging confirmed colitis despite mild GI symptoms, guiding treatment	[7]
Antibiotic management	Early empiric therapy guided by local resistance, de-escalation once susceptibilities known	Piperacillin-tazobactam empiric, narrowed to levofloxacin based on culture	Optimizes treatment, minimizes unnecessary broad-spectrum antibiotic exposure, and supports antimicrobial stewardship	[1,3]

## Discussion

Shigellosis is classically associated with inflammatory diarrheal symptoms such as bloody or mucoid stools, tenesmus, and frequent small-volume bowel movements [1]. While many infections are self-limited, severe disease can occur and may require hospitalization, particularly when systemic features are present [1].

In contrast to typical presentations, our patient reported only non-bloody diarrhea and denied tenesmus, instead presenting with nonspecific systemic symptoms including generalized weakness, abdominal pain, and low-grade fever. Stool cultures confirmed *Shigella sonnei* (group D), the predominant serogroup in the United States [4]. The absence of classic dysenteric features, combined with communication barriers, contributed to diagnostic complexity and delayed recognition. Similar atypical presentations have been described in adult cases of *Shigella* colitis and bacteremia, in which symptoms were initially attributed to noninfectious causes before confirmatory testing established the diagnosis [8,9]. These reports, together with the present case, highlight the importance of maintaining a broad differential diagnosis for adults presenting with systemic symptoms and abdominal complaints, even in the absence of overt dysentery.

Objective laboratory and imaging findings were critical in guiding clinical decision-making. Although gastrointestinal symptoms were relatively mild, the presence of fever, leukocytosis (11.7 × 10⁹/L), and elevated lactate (3.9 mmol/L) suggested a systemic inflammatory response and prompted further evaluation. Cross-sectional imaging demonstrated segmental colonic wall thickening with pericolonic inflammatory changes, confirming acute colitis and supporting the initiation of empiric broad-spectrum antimicrobial therapy. While the lactate elevation was modest, it served as an early indicator of physiologic stress and contributed to the escalation of diagnostic and therapeutic interventions. This case demonstrates that laboratory and imaging indicators can provide actionable guidance, even when classic clinical signs are absent.

Recent methamphetamine use complicated history-taking and may have influenced early clinical assessment. Although substance use did not directly contribute to the development of *Shigella* enterocolitis, cognitive biases such as anchoring or premature closure can occur when symptoms are attributed to intoxication or withdrawal rather than underlying infection [10,11]. Prior studies on diagnostic error in emergency and acute care settings emphasize that reliance on objective diagnostic data - including laboratory testing, imaging, and microbiologic studies - can mitigate these biases and reduce delayed or missed diagnoses [10,11]. In this case, early diagnostic evaluation facilitated the timely identification of *S. sonnei *despite an initially nonspecific presentation, highlighting the role of structured diagnostic approaches in improving patient safety.

Management of shigellosis is further complicated by rising antimicrobial resistance, with increasing resistance to fluoroquinolones, azithromycin, and third-generation cephalosporins, as well as the emergence of extensively drug-resistant strains in the United States [2,10,11]. Current guidelines recommend empiric antibiotic therapy for severe or hospitalized infections, tailored to local susceptibility patterns, with prompt de-escalation once culture results are available [2,12]. In our patient, empiric broad-spectrum therapy was appropriately narrowed to oral levofloxacin based on susceptibility data, reflecting antimicrobial stewardship principles [12]. This approach optimized patient care, minimized unnecessary broad-spectrum antibiotic exposure, and contributed to responsible management of rising resistance trends.

Recent epidemiologic data further emphasize the clinical relevance of severe adult shigellosis. Surveillance data show that approximately 28% of adult *Shigella* infections meet criteria for severe disease - including hospitalization, bacteremia, or death - with the highest incidence among African American males aged 18-49 years [13]. These findings demonstrate that severe shigellosis in adults is neither rare nor confined to traditionally recognized high-risk groups. Accordingly, cases such as ours highlight the need for timely diagnostic evaluation, including stool cultures and, when indicated, imaging, in adults presenting with unexplained systemic symptoms and abdominal pain.

This report has several limitations inherent to single-patient case descriptions. The analysis remains descriptive and cannot establish causal relationships or quantify the prognostic significance of individual indicators such as elevated lactate or systemic inflammatory features. Serial laboratory measurements were unavailable, limiting assessment of biomarker kinetics and response to therapy. Additionally, microbiologic susceptibility data were reported qualitatively without minimum inhibitory concentration values, and post-discharge follow-up was unavailable due to loss to follow-up. Despite these limitations, this case highlights clinically important principles, including recognition of atypical *Shigella* presentations, the value of objective diagnostic testing in mitigating cognitive bias, and the importance of timely antibiotic de-escalation. While hypothesis-generating rather than definitive, these observations may inform diagnostic vigilance and antimicrobial stewardship in hospitalized adult patients.

## Conclusions

This case of *Shigella* enterocolitis highlights the need for diagnostic flexibility when evaluating adult patients with atypical or nonspecific symptoms, particularly in the setting of substance use or unreliable history. Early utilization of objective diagnostic tools - including imaging and stool cultures - was critical for timely identification and guided targeted antimicrobial therapy. In the context of rising antimicrobial resistance among *Shigella* species, clinicians should prioritize empiric antibiotic selection informed by local susceptibility patterns, promptly de-escalate therapy based on culture results, and adhere to antimicrobial stewardship principles. Beyond individual patient care, recognition and reporting of such cases contribute to broader public health surveillance and inform strategies to mitigate the spread of multidrug-resistant and extensively drug-resistant *Shigella*. Ultimately, maintaining a high index of suspicion for infectious etiologies, even when presentations deviate from classical patterns, can improve clinical outcomes and support evidence-based management in complex adult cases.

Adult shigellosis can present atypically, without classic dysenteric features. Early objective testing, culture-guided therapy, and adherence to stewardship principles are essential to optimize outcomes and limit the impact of rising antimicrobial resistance.
